# Dietary Starch–Extract Complexes from Cerrado Fruits Modulate Oxidative Stress in Mononuclear Cells from Normoglycemic and Diabetic Individuals

**DOI:** 10.3390/antiox15010044

**Published:** 2025-12-29

**Authors:** Paula Becker Pertuzatti, Karielly Pereira Montel, Priscila Delalibera, Yasmin Aparecida Konda-Barros, Viviane Francelina Luz, Adenilda Cristina Honório-França, Eduardo Luzia França, Ricardo Stefani, Danilo Hiroshi Konda

**Affiliations:** 1 Programa de Pós-Graduação em Imunologia e Parasitologia Básicas e Aplicadas, Instituto de Ciências Biológicas e da Saúde, Universidade Federal de Mato Grosso, Avenida Valdon Varjão, 6.390, Barra do Garças 78605-091, MT, Brazil; karielly.montel@sou.ufmt.br (K.P.M.); viviane.luz@sou.ufmt.br (V.F.L.); adenilda.franca@ufmt.br (A.C.H.-F.); eduardo.franca@ufmt.br (E.L.F.); 2 Programa de Pós-Graduação em Ciência de Materiais, Instituto de Ciências Exatas e da Terra, Universidade Federal de Mato Grosso, Avenida Valdon Varjão, 6.390, Barra do Garças 78605-091, MT, Brazil; priscila.oliveira@sou.ufmt.br (P.D.); ricardo.stefani@ufop.edu.br (R.S.);; 3 Instituto de Ciências Biológicas e da Saúde, Universidade Federal de Mato Grosso, Avenida Valdon Varjão, 6.390, Barra do Garças 78605-091, MT, Brazil; 4 Laboratório de Polímeros, Inteligência Artificial e Produtos Naturais, Departamento de Química, Instituto de Ciências Exatas e Biológicas, Universidade Federal de Ouro Preto, Ouro Preto 35400-000, MG, Brazil

**Keywords:** bioactive compounds, reactive oxygen species (ROS), *Solanum lycocarpum*, *Buchenavia tomentosa* Eichler, non-conventional starch, starch characterization, X-ray diffraction, type 2 diabetes, immunomodulatory effect

## Abstract

Cerrado fruits are rich sources of bioactive compounds with antioxidant and immunomodulatory properties. However, it remains unclear whether the complexes of non-conventional starch with extracts from these fruits can modulate oxidative stress in human cells, under diabetic conditions. This study evaluated the effects of lobeira (*Solanum lycocarpum*) starch complexed with hydrophilic and lipophilic extracts of mirindiba (*Buchenavia tomentosa*) on redox parameters in mononuclear cells from normoglycemic and diabetic individuals. The extracts showed high phenolic (1362.70 mg gallic acid equivalent (GAE)/100 g) and carotenoid content (7.07 mg β-carotene/100 g) and strong antioxidant capacity (58.42–140.19 μmol Trolox/g by FRAP and DPPH). Structural analyses (Fourier transform infrared (FTIR), X-ray diffraction (XRD), and Scanning Electron Microscopy (SEM)) confirmed complexation via hydrogen bonding and inclusion-type interactions, which partially modified the crystalline order of starch. The complexes exhibited high biocompatibility (>97% cell viability) and adaptively modulated oxidative and antioxidant responses under different metabolic and infectious conditions. Normoglycemic cells showed enhanced redox balance, with moderate superoxide generation and higher SOD activity, while cells from diabetic individuals displayed elevated oxidative stress and reduced SOD induction upon treatment. Under the *E. coli* challenge, the complexes modulated redox equilibrium through compensatory antioxidant responses. These findings position lobeira starch–mirindiba extract complexes as promising dietary immunomodulators against oxidative stress in metabolic and infectious contexts.

## 1. Introduction

The Brazilian Cerrado is one of the world’s richest biomes in terms of biodiversity; however, much of this potential remains underexplored [1,2]. Among its native fruits, pequi (*Caryocar brasiliense* Camb.), mangaba (*Hancornia speciosa*), and cajuzinho-do-Cerrado (*Anacardium humile*) have already shown immunomodulatory effects associated with their content of bioactive compounds such as phenolics and carotenoids, particularly through modulation of oxidative stress markers, including superoxide anion, superoxide dismutase, and the oxidative index [3,4,5]. In addition, buriti (*Mauritia flexuosa*) extracts preserve the viability of human mononuclear cells challenged with enteropathogenic *Escherichia coli* (EPEC), further reinforcing the immunomodulatory potential of Cerrado fruits [6]. In addition to being valuable sources of phenolics and carotenoids, certain Cerrado fruits, such as lobeira (*Solanum lycocarpum*), also stand out as non-conventional starch sources [7], opening new perspectives for incorporating bioactive compounds into starch matrices.

Starch is composed of amylose and amylopectin, with the former contributing to amorphous regions and the latter to crystalline regions, whose organization defines the A-, B-, and C-type crystallinity patterns [8]. In recent years, few studies have investigated the complexation of conventional starches, such as rice and maize, with phenolic compounds and, to a lesser extent, with carotenoids. In both cases, the complexation resulted in the formation of V-type inclusion complexes, which can inhibit enzymatic hydrolysis by α-amylase and α-glucosidase, thereby reducing digestibility and consequently the glycemic index [9,10,11]. This approach also alters physicochemical properties, such as thermal stability and crystallinity, as well as biological characteristics, including digestibility and antioxidant capacity, thus broadening its potential applications at the food-health interface.

Although starch–bioactive complexes show promising applications at the food-health interface, their biological effects remain poorly understood, as, to the best of our knowledge, no studies have reported their evaluation in cellular or animal models. Investigations into digestibility, resistant starch formation, and inhibition of amylolytic enzymes suggest potential benefits for individuals with diabetes. Diabetes, the most prevalent metabolic disorder worldwide, is characterized by chronic hyperglycemia that triggers oxidative stress through glucose auto-oxidation and mitochondrial dysfunction, leading to excessive radical accumulation and reduced antioxidant defenses [12]. Although dietary antioxidants have been widely studied in the context of diabetes [13,14,15,16], it remains unclear whether starch–extract complexes interfere with these mechanisms, particularly in cells from individuals with diabetes. In this context, it remains unknown whether complexes formed from non-conventional starches and Cerrado fruit extracts can induce structural changes that modulate oxidative stress in human cells. Therefore, we hypothesized that the complexation of lobeira starch (*Solanum lycocarpum*) with mirindiba extracts (*Buchenavia tomentosa* Eichler) rich in phenolic compounds and carotenoids induces structural modifications that modulate reactive species in mononuclear cells, particularly under diabetic conditions.

In this context, the present study aimed to investigate the effects of lobeira (*S. lycocarpum*) starch complexed with hydrophilic and lipophilic extracts of mirindiba (*B. tomentosa*). Particular attention was given to the bioactive compound profile and antioxidant capacity of the extracts, as well as to the structural modifications induced by the complexation. Redox parameters were then assessed in mononuclear cells from normoglycemic and type 2 diabetic individuals. Altogether, this approach seeks to clarify whether the complexation of non-conventional starch with bioactive extracts from Cerrado fruits may induce structural modifications and modulate oxidative balance under distinct metabolic conditions.

## 2. Materials and Methods

### 2.1. Raw Material and Reagents

The fruits of lobeira (*S. lycocarpum*) were harvested unripe (5 kg) in September 2024 in the municipality of Pontal do Araguaia (15°50′39″ S, 52°0′13″ W), Mato Grosso, Brazil. Meanwhile, mirindiba fruits (*B. tomentosa*) were collected ripe (2 kg) during the same period in the municipality of Barra do Garças (15°53′35″ S, 52°15′36″ W), Mato Grosso, Brazil. The fruits were selected, sanitized, and stored in vacuum-sealed high-density polyethylene (HDPE) plastic bags, then kept frozen at −18 °C until analysis.

The chemicals gallic acid (97.5–102.5%), caffeic acid (98%), (±)-6-hydroxy-2,5,7,8-tetramethylchroman-2-carboxylic acid (Trolox^®^, 97%), 2,2-diphenyl-1-picrylhydrazyl (DPPH), acridine orange, cytochrome C, and nitroblue tetrazolium were purchased from Sigma-Aldrich (St. Louis, MO, USA). Ficoll-Paque was obtained from Pharmacia (Uppsala, Sweden), quercetin (98%) from Cayman Chemical Company (Ann Arbor, MI, USA), and 2,4,6-tris(2-pyridyl)-s-triazine (TPTZ, 98%) from Berkshire (London, UK).

### 2.2. Proximate Composition of Lobeira (S. lycocarpum)

The moisture content (AOAC 934.01), proteins (AOAC 2001.11), lipids (AOAC 920.39), and ash (AOAC 942.05) were determined according to the procedures described by the Association of Official Analytical Chemists [17]. Carbohydrate content was determined by subtracting the sum of moisture, protein, lipids, and ash from 100. The energetic value was calculated using Atwater conversion factors (4 kcal/g for proteins and carbohydrates, and 9 kcal/g for lipids).

### 2.3. Starch Extraction

Lobeira starch was extracted following an aqueous extraction method with slight modifications [18]. Unripe lobeira fruits were cut, weighed, and blended with distilled water in a 1:2 (*w*/*v*) ratio using a household blender (Philco, model PH900 turbo, São Paulo, Brazil) at room temperature until a homogeneous mixture was obtained. The resulting suspension was refrigerated at 4 °C for 24 h to allow starch hydration. After this period, the material was homogenized again (without adding more water) and subjected to static decantation for an additional 24 h under the same temperature conditions (4 °C). The decanted material was filtered and sieved through an 88-mesh screen. The filtrate was purified through three successive washes with distilled water. In each step, the suspension was homogenized using a vortex mixer (Norte Científica, São Paulo, Brazil, model NA 3600, 28 Hz) and centrifuged at 3000 rpm for 15 min, with the supernatant discarded after each wash. The residue was then treated with absolute ethyl alcohol (100 mL per 500 g of initial material), followed by vacuum filtration. The retained starch was dried in a forced-air oven at 40 °C for 24 h, cooled in a desiccator, carefully removed, and stored in tightly sealed containers protected from light. The starch yield was calculated as the ratio between the initial fruit mass and the final mass of dried crude starch and expressed as a percentage (%). In addition, the total phenolic content of the purified lobeira starch was determined in order to verify the presence of residual phenolic compounds after extraction and purification. The procedure used for phenolic quantification is described in Section 2.5.1.

### 2.4. Preparation of Mirindiba Extracts (B. tomentosa)

Two types of mirindiba extracts were obtained, characterized for their bioactive compound content, and subsequently used to produce the starch–extract complexes. For extract preparation, the seeds were removed, and the remaining material (peel and pulp) was ground.

The hydrophilic extract was prepared according to the procedure described by Barcia et al. [19]. Approximately 5 g of ground mirindiba was extracted with 25 mL of a methanol/water/formic acid (50:48.5:1.5) solution for 15 min in an ultrasonic bath (Arruda, Ultrassons LTDA, Indaiatuba, Brazil). The mixture was filtered through filter paper, and the extraction was repeated once. The final extract (50 mL) was stored in the dark at room temperature until use. The hydrophilic extract was used for determining total phenolic compounds, total flavonoids, total phenolic acids, and antioxidant capacity.

The lipophilic extract of mirindiba was obtained according to the adapted method of Rodriguez-Amaya [20]. Briefly, 5 g of whole fruit (peel and pulp, seeds removed) was homogenized with 20 mL of acetone using an Ultra-Turrax (Marconi, model MA 102/Plus, Piracicaba, Brazil) for 1 min and filtered under vacuum. This procedure was repeated five times to ensure exhaustive carotenoid extraction. The combined acetone fractions were transferred to a separation funnel, mixed with petroleum ether, and partitioned, then washed with distilled water to remove hydrophilic residues. The lipophilic phase was collected and adjusted to a final volume of 50 mL in a volumetric flask. This extract was used for both carotenoid quantification and starch complexation assays.

### 2.5. Characterization of Mirindiba Extracts

#### 2.5.1. Bioactive Compounds

Total phenolic content (TPC) was quantified using the Folin–Ciocalteu method adapted from Singleton et al. [21]. Briefly, 500 μL of hydrophilic extract was mixed with 2.5 mL of Folin–Ciocalteu reagent (0.2 mol/L). After 5 min, 2 mL of a sodium carbonate solution (75 g/L) was added, and the mixture was kept in the dark for 2 h. Absorbance was measured at 760 nm using a spectrophotometer, and a standard curve of gallic acid (0.01–0.08 mg/mL) was used. Results were expressed as milligrams of gallic acid equivalent per 100 g of sample (mg GAE/100 g of fresh sample).

Total flavonoid content (TFC) was determined following the aluminum chloride colorimetric method [22]. Equal volumes of hydrophilic extract (5 mL) and 2% aluminum chloride solution in methyl alcohol (*m*/*v*) were combined and incubated in the dark for 30 min. Absorbance was measured at 415 nm against a quercetin calibration curve (2.5–20 mg/L). Data were expressed as milligrams of quercetin equivalent per 100 g of sample (mg QE/100 g of fresh sample).

Total phenolic acid content (TPAC) was measured according to the method of Mazza et al. [23] with modifications. A mixture containing 0.25 mL of hydrophilic extract, 0.25 mL of 0.1% HCl in 95% ethyl alcohol (*v*/*v*), 4.5 mL of 2% HCl (*v*/*v*), and 5 mL of distilled water was incubated in the dark for 15 min. Absorbance was measured at 320 nm using a caffeic acid standard curve (15–200 mg/L). Results were expressed as milligrams of caffeic acid equivalent per 100 g of sample (mg CAE/100 g of fresh sample).

Total carotenoid content (TCC) was determined by direct spectrophotometric measurement of lipophilic extracts at 450 nm, prepared according to Rodriguez-Amaya [20]. The absorption coefficient of β-carotene in petroleum ether (2592) was used for quantification. Data were expressed as milligrams of β-carotene per 100 g of sample (mg β-carotene/100 g of fresh sample).

Chlorophyll content was quantified following the spectrophotometric procedure described by Porra et al. [24], with adaptations. Absorbance readings were recorded at 663, 646, and 750 nm, corresponding to chlorophyll *a*, *b*, and total chlorophyll (*a* + *b*), respectively. Calculations were performed using the specific equations proposed by the authors, and results were expressed as mg of chlorophyll per 100 g of sample (mg chlorophyll/100 g of fresh sample).

#### 2.5.2. Antioxidant Capacity

The radical-scavenging capacity against DPPH was evaluated according to the method of Brand-Williams et al. [25]. Aliquots of 100 μL of hydrophilic extract were mixed with 3.9 mL of DPPH working solution in methyl alcohol (prepared to an absorbance of 1.1 ± 0.2 at 517 nm, equivalent to ~0.06 mmol/L) and incubated for 30 min in the dark. Absorbance was measured at 517 nm in a spectrophotometer (Kasuaki UV-VIS, São Paulo, Brazil). Quantification was based on a Trolox calibration curve (50–1500 μmol/L). Results were expressed as μmol Trolox equivalent per 100 g of sample (μmol Trolox/100 g of fresh sample).

The Ferric-Reducing Antioxidant Power (FRAP) assay was performed according to the method of Benzie and Strain [26]. Reaction mixtures contained 2400 μL of FRAP solution, 240 μL of distilled water, and 80 μL of hydrophilic extract. After incubation in a water bath at 37 °C for 15 min, absorbance was measured at 593 nm. A Trolox calibration curve (100–500 μmol/L) was used for quantification. Results were expressed as μmol Trolox equivalent per 100 g of sample (μmol Trolox/100 g of fresh sample).

### 2.6. Lobeira Starch Complexation

#### 2.6.1. Preparation of the Starch–Hydrophilic Extract Complex

The complexation of lobeira starch with the hydrophilic extract of mirindiba was performed according to the method of Amoako and Awika [27], with adaptations. 50 mL of hydrophilic extract, obtained as described in Section 2.4, was rotary evaporated to 20 mL and then added to 8 g of starch in a beaker, obtained as described in Section 2.3. The final volume was adjusted to 80 mL with 30% aqueous ethanol, yielding a suspension totaling 100 mL. Gelatinization was performed in a water bath at 70 °C for 30 min under mechanical stirring (1000 rpm). After cooling to 30 °C under constant agitation, the suspension was transferred into 15 mL Falcon tubes and centrifuged at 4000 rpm for 5 min. Pellets were washed twice with 3 mL of absolute ethanol. After each wash, the samples were vortexed to ensure homogenization and then centrifuged at 4000 rpm for 5 min to remove unbound compounds. The precipitates were dried overnight at 40 °C, finely ground, and stored at 4 °C until analysis.

#### 2.6.2. Preparation of the Starch–Lipophilic Extract Complex

Complexation with lipophilic extract followed the procedure of Song et al. [11], with adaptations. Starch (8 g), obtained as described in Section 2.3, was dispersed in 95 mL of distilled water. 50 mL of lipophilic extract, obtained as described in Section 2.4, was rotary-evaporated to remove the solvent, then resuspended in 5 mL of acetone. Starch gelatinization was carried out in a water bath at 70 °C for 30 min under mechanical stirring (1000 rpm). At the beginning of gelatinization, the lipophilic extract solution was added dropwise to the starch suspension using a pipette with extended tips to reach the sample surface. After cooling to 30 °C under continuous stirring, the mixture was aliquoted into 15 mL Falcon tubes and centrifuged at 4000 rpm for 5 min. Pellets were washed twice with 3 mL of absolute ethanol; after each wash, samples were vortexed and centrifuged (4000 rpm, 5 min) to remove unbound compounds. The precipitates were dried at 40 °C (overnight), finely ground to achieve a uniform particle size, and stored at 4 °C until further use.

### 2.7. Characterization of Starch and Starch–Extract Complexes

#### 2.7.1. Fourier Transform Infrared (FT-IR) Spectroscopy

FTIR spectra were recorded using a PerkinElmer Spectrum (PerkinElmer Inc., Waltham, MA, USA) (model spectrum 100) equipped with an attenuated total reflectance (ATR) accessory with a germanium (Ge) crystal. Measurements were carried out over the range 4000–600 cm^−1^, with a resolution of 4 cm^−1^. This analysis was performed to identify the main functional groups of lobeira starch and its complexes with mirindiba extracts.

#### 2.7.2. Scanning Electron Microscopy (SEM)

The starch samples were sputter-coated with a thin layer of gold using a DII-29010SCTR Smart Coater (JEOL Ltd., Tokyo, Japan). Subsequently, the samples were examined in a VEGA3 scanning electron microscope (TESCAN, Brno, Czech Republic). Analyses were performed under high-vacuum conditions (HV) at an accelerating voltage of 5.0 kV. The working distance (WD) was maintained at 4.66 mm, corresponding to the distance between the sample surface and the lower edge of the objective lens. Images were obtained at magnifications of 50×, 100×, and 500×, with fields of view ranging from 159 to 182 μm.

#### 2.7.3. X-Ray Diffraction (XRD)

X-ray diffraction (XRD) patterns of lobeira starch samples were obtained using a diffractometer operating with Cu Kα radiation, operating at 40 kV and 15 mA. Data were collected over a 2θ range of 5–50°, with a step size of 0.02°/s and a total acquisition time of 50 min. The resulting diffractograms were analyzed to determine the crystalline patterns and degree of crystallinity of the starch and starch–extract complexes.

#### 2.7.4. Thermogravimetric Analysis (TG) and Differential Scanning Calorimetry (DSC)

Thermal analyses were performed using a Mettler Toledo thermal analyzer (Mettler-Toledo GmbH, Greifensee, Switzerland) (model TG-DS-1). Approximately 4 mg of each sample was placed in alumina open crucibles (70 μL capacity) and heated from 30 °C to 1000 °C at 20 °C/min under a dry air flow of 60 mL/min. TG curves were used to evaluate weight loss profiles, while DSC curves provided information on thermal transitions and stability.

### 2.8. Biological Activity of Starch–Extract Complexes

#### 2.8.1. Subjects

The study included 10 volunteers (five normoglycemic and five with type 2 diabetes), both men and women, aged 40–70 years, all non-smokers. From each subject, 10 mL of peripheral blood was collected in EDTA tubes. The study was conducted in accordance with the Declaration of Helsinki and approved by the Ethics Committee of the Federal University of Mato Grosso, Araguaia Campus (CAAE protocol no. 27068719.1.0000.5587). Written informed consent was obtained from all participants before sample collection.

#### 2.8.2. Isolation of Mononuclear Cells (MN)

Peripheral blood mononuclear cells (MN) were isolated using a Ficoll-Paque gradient, yielding preparations with 95% purity, verified by light microscopy, following the procedure adapted from Pessoa et al. [28]. Blood was collected once from each participant, and all experimental treatments with starch and starch–extract complexes were performed ex vivo on the isolated MN; No additional post-treatment blood collection was performed. Briefly, blood samples were layered onto Ficoll and centrifuged for 40 min at 1500 rpm at room temperature (25 °C). The mononuclear cell ring formed at the interface was carefully collected with a Pasteur pipette and washed twice with 3 mL of phosphate-buffered saline (PBS). After each wash, the supernatant was discarded and the pellet resuspended. Finally, cells were suspended in 1 mL of PBS, counted in a Neubauer chamber, and adjusted to a final concentration of 2 × 10^6^ cells/mL.

#### 2.8.3. Cell Viability

Cell viability was assessed by acridine orange staining, following the protocol of Bellinate-Pires et al. [29]. Briefly, 500 μL of freshly isolated peripheral blood mononuclear cells (MN) were incubated in a water bath at 37 °C with 250 μL of aqueous suspensions of native starch or starch–extract complexes at final concentrations of 100 μg/mL, 100 ng/mL, or 100 pg/mL for 30 min. After incubation, the suspensions were centrifuged at 1500 rpm for 10 min, the supernatant was discarded, and the pellet was stained with 200 μL of acridine orange solution (14.4 mg/mL) for 1 min. Cells were resuspended and washed twice with PBS before mounting on slides. A total of 100 cells were counted under a fluorescence microscope (Nikon Eclipse E-200, Nikon Corporation, Tokyo, Japan) and classified as viable when exhibiting green fluorescence and non-viable when showing orange fluorescence. Cell viability was expressed as the percentage of viable cells relative to the total counted.

#### 2.8.4. *Escherichia coli* Strain and Incubation with MN Cells

The enteropathogenic *Escherichia coli* (EPEC) was used to assess the functional activity of blood MN cells. This bacterium was isolated from the stool of an infant with acute diarrhea (serotype 0111:H AL^−^, *eae*^+^, *eaf*^+^, *bfp*^+^). The EPEC was prepared and adjusted to 10^7^ bacteria/mL.

For the assays, equal volumes of MN cells, starch or starch–extract complex suspensions (final concentration: 100 ng/mL), and the EPEC culture were incubated together in a water bath at 37 °C for 30 min. The negative control consisted of MN cells incubated with EPEC in the absence of starch or complexes. These preparations were subsequently used to determine superoxide anion production, superoxide dismutase (SOD) activity, and the oxidative index.

#### 2.8.5. Superoxide Anion Production (O_2_^•−^)

The release of superoxide anion (O_2_^•−^) by MN cells, in the presence or absence of EPEC, was measured according to the cytochrome C reduction method adapted from Pick and Mizel [30]. MN cells previously incubated with starch, starch–extract complexes (100 ng/mL), and/or EPEC (as described in Section 2.8.4) were centrifuged (1500 rpm, 10 min) and resuspended in PBS containing 2.6 mmol/L CaCl_2_, 2 mmol/L MgCl_2_, and cytochrome C. Samples were further incubated at 37 °C for 24 h in the dark. Absorbance was measured at 540 nm using a spectrophotometer (Thermo Plate TP-Reader, Thermo Plate Ltda., São Paulo, Brazil). Results were expressed as nmol O_2_^•−^. All experiments were performed in duplicate.

#### 2.8.6. Superoxide Dismutase (SOD) Activity

SOD activity was assessed using the nitroblue tetrazolium (NBT) reduction method. Supernatants from MN cells incubated with starch or starch–extract complexes (100 ng/mL), with or without EPEC, were mixed with 500 μL of a chloroform–ethanol mixture (1:1, *v*/*v*), 500 μL of the NBT-EDTA reagent mixture (1:1.5, *v*/*v*), and 2 mL of hydroxylamine buffer. After incubation, absorbance was read at 560 nm, and results were expressed as international units (IU) of CuZn-SOD activity.

#### 2.8.7. Oxidative Stress Index (OI)

The oxidative stress index (OI) was calculated to integrate the balance between pro-oxidant and antioxidant parameters. The OI was obtained as the ratio between CuZn-SOD activity and superoxide anion concentration, according to the equation

(1) $$OI=\frac{Active\;SOD}{\left[{O_{2}}^{•-}\right]}$$

where *Active SOD* corresponds to the enzymatic activity determined in UI and [O_2_^•−^] represents the concentration of superoxide anion measured in the assays.

### 2.9. Statistical Analysis

The results were expressed as mean ± standard deviation. Data normality was verified using the Shapiro–Wilk test, and homoscedasticity was assessed with Levene’s test. When both assumptions were met, the data were subjected to Analysis of Variance (ANOVA) followed by Tukey’s post hoc test to evaluate differences among groups, considering a significance level of 5%.

## 3. Results and Discussion

### 3.1. Proximate Composition of Lobeira

The proximate composition of lobeira fruit (Table 1) revealed that carbohydrates were the predominant dry matter component, highlighting the fruit as a potential non-conventional starch source. As expected for fresh fruits, lobeira also exhibited a high moisture content, while lipids and ash were minor components.

The lobeira starch yield reached 55.55% (db), a value superior to that reported for several unconventional sources such as immature apples (23–28%, db), kiwifruit (40%, db), pineapple stems (30%, db), and annatto seeds (18–20%, db), though slightly below that of classic starchy fruit like unripe bananas (70–80%) [31,32]. This intermediate profile positions lobeira as a competitive alternative source, reinforcing its potential for valorization in the food and pharmaceutical sectors, which represents an opportunity to supply the growing industrial demand for starches with distinct properties and to diversify raw material sources. Altogether, these findings support the use of lobeira starch as a sustainable raw material for the development of starch–bioactive complexes with potential health applications.

### 3.2. Bioactive Compounds of Mirindiba

The mirindiba hydrophilic extract exhibited a high total phenolic content (TPC) of 1362.70 ± 33.49 mg GAE/100 g fresh sample. Within this phenolic pool, total phenolic acids accounted for 73.86 ± 0.52 mg CAE/100 g fresh sample, and total flavonoids were quantified at 100.14 ± 1.80 mg QE/100 g fresh sample, representing only about 13% of the total phenolics. This disparity suggests that additional subclasses of phenolic constituents, such as hydrolysable tannins, proanthocyanidins, and phenolic glycosides remain unquantified. However, because the Folin–Ciocalteu assay is sensitive not only to phenolics but also to reducing non-phenolic compounds (e.g., sugars, ascorbate, peptides, and some minerals), the difference between TPC values and the quantified subclasses should be interpreted with caution. This gap may indeed reflect the presence of other phenolic families, but it could also arise from contributions of various reducing compounds present in the extract. In studies with acetone extracts of mirindiba leaves, gallic acid, ellagic acid, epicatechin, kaempferol, vitexin, and corilagin have already been identified, confirming the chemical diversity of this species and reinforcing the likelihood that similar or derivative compounds are present in the fruit matrix [33].

When compared to other Cerrado fruits, mirindiba stands out for its significantly higher phenolic content, greatly exceeding values reported for pequi (*Caryocar brasiliense* Camb.) (54.60 mg GAE/100 g) [3], cajuzinho-do-Cerrado (*Anacardium humile*) (189.80 mg GAE/100 g) [5], and murici (*Byrsonima verbascifolia*) (303.50 mg GAE/100 g) [34]. This substantial phenolic content positions *B. tomentosa* as one of the most phenolic-rich and biologically relevant antioxidant sources within the Cerrado biome, though its levels remain within the expected range for phenolic-rich plant species more broadly. In addition, the purified lobeira starch used for complexation exhibited a low total phenolic content (72.87 ± 0.44 mg GAE/100 g), more than one order of magnitude lower than that of the mirindiba hydrophilic extract. This confirms that the phenolic content and antioxidant-related effects observed in the starch–extract complexes predominantly originate from mirindiba bioactives rather that residual phenolics associated with starch matrix.

Beyond its high concentration of hydrophilic compounds, *B. tomentosa* also exhibits substantial levels of lipophilic bioactive compounds. Among these, carotenoids were quantified at 7.07 ± 1.22 mg β-carotene/100 g fresh sample, while significant amounts of chlorophylls a (0.42 ± 0.04 mg/100 g fresh sample), chlorophyll b (0.83 ± 0.02 mg/100 g fresh sample), and total chlorophyll (1.25 ± 0.02 mg/100 g fresh sample) were also detected. Cerrado fruits are well recognized for their high content of carotenoids, bioactive molecules known for their ability to attenuate oxidative stress. Previous studies on *B. tomentosa* have identified α-carotene, β-carotene, and lycopene in the fruit, with higher concentrations observed in the peel than in the pulp [35].

Given the high concentrations of carotenoids and phenolic compounds in mirindiba, both well-documented antioxidant classes, mirindiba extracts showed strong antioxidant capacity. The mirindiba extract displayed 140.19 ± 2.67 μmol Trolox/g fresh sample for DPPH and 58.42 ± 4.34 μmol Trolox/g fresh sample for FRAP, confirming its substantial radical-scavenging and reducing abilities. The higher capacity observed in the DPPH method reflects the predominance of compounds capable of donating hydrogen atoms or electrons to neutralize free radicals, involving both hydrogen atom transfer (HAT) and single-electron transfer (SET) mechanisms. This mechanistic profile is consistent with observations in other Cerrado fruit extracts rich in mixed phenolic-carotenoid systems, where phenolics contribute mainly through radical-chain interruption and carotenoids through physical quenching rather than ferric reduction [3,6], reinforcing the expected antioxidant behavior observed here.

These findings highlight that *B. tomentosa* bioactives operate through complementary antioxidant mechanisms, integrating radical scavenging and redox modulation. The overall high antioxidant capacity reinforces the synergistic potential of hydrophilic and lipophilic constituents and supports their application in starch–bioactive complexes designed to modulate oxidative stress.

### 3.3. Characterization of Starch and Starch–Extract Complexes

#### 3.3.1. Fourier Transform Infrared (FT-IR) Spectroscopy

**Figure 1:**
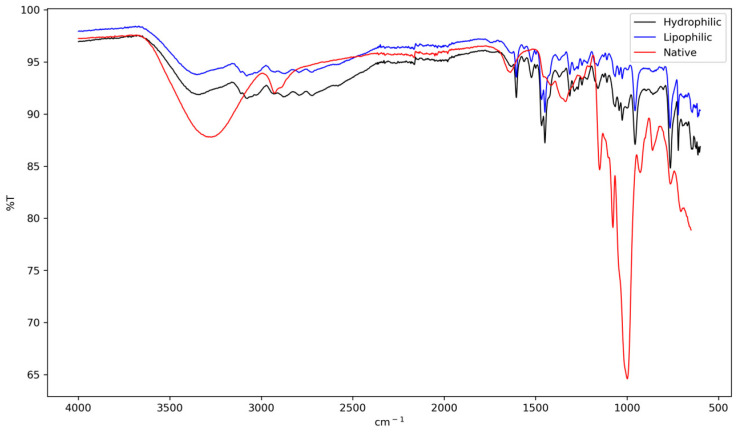
FTIR spectra of native lobeira (*S. lycocarpum*) starch, starch complexed with hydrophilic extract of mirindiba (*Buchenavia tomentosa* Eichler), and starch complexed with lipophilic extract of mirindiba (*Buchenavia tomentosa* Eichler).

FTIR analysis (Figure 1) revealed distinct noncovalent interactions between the extract components and starch matrix. The native starch, used as a control, displayed its characteristic spectrum: a broad band centered at ~3400 cm^−1^ (O-H stretching), peaks at ~2930 cm^−1^ (C-H stretching), a band at ~1644 cm^−1^ (H-O-H bending of bound water), and the fingerprint region (1200–1000 cm^−1^), associated with C-O and C-O-C vibrations of the polysaccharide structure [36,37,38].

The broadening and shift of the O-H band to a lower wavenumber (~3385 cm^−1^) indicate the formation of a more diverse and extensive hydrogen-bonding network between the hydroxyl groups of starch and those of phenolic compounds present in the hydrophilic extract [39]. This interaction suggests a strong affinity between the two components, potentially altering the physicochemical properties of the starch.

Further evidence of structural reorganization is provided by the decrease in the 1047/1022 cm^−1^ absorbance band, which serves as a short-range molecular order index [40,41]. This reduction, compared to native starch, implies a partial disruption of the original crystalline structure. Such modifications in starch structure, induced by the presence of phenolic compounds, have been linked to changes in enzymatic digestibility [18]. This relationship between structural alterations and digestibility has significant implications for potential applications in food science and nutrition, as it may influence the glycemic response and overall digestive properties of starch-based products containing phenolic-rich extracts.

In contrast, the complex containing the lipophilic extract exhibited a marked intensification of the bands ~2925 and ~2854 cm^−1^, confirming the incorporation of aliphatic chains. Concurrently, the relative intensity of the O-H band decreased. This set of spectral features strongly suggests the formation of V-type amylose inclusion complexes [39]. These complexes, in which lipid chains are hosted within the amylose helix, are known to promote new molecular arrangements that enhance thermal stability and reduce hydrolysis [38].

Additionally, the spectral differences observed in the 1000–700 cm^−1^ region confirm the structural changes occurring during the formation of starch–bioactive complexes. Native starch sharper and more defined peaks in this region are indicative of its well-organized glycosidic linkages and helical arrangements. In contrast, the smoother and less intense bands exhibited by both hydrophilic and lipophilic complexes suggest a disruption of this original structure. This alteration points to a reduction in short-range molecular order and a partial reorganization of the amylose helical structure, a behavior consistent with previous reports showing that interactions with phenolic compounds can disrupt native starch ordering through hydrogen bonding and inclusion-type interactions [42].

These spectral changes, combined with other analytical results such as SEM and XRD, offer strong evidence for the interaction between phenolic and carotenoid-rich extracts and the starch matrix. The mechanism of this interaction involves both hydrogen bonding and hydrophobic inclusion, leading to significant molecular rearrangements within the starch structure. This complex formation process not only alters the physical properties of the starch but also potentially enhances the stability and bioavailability of the incorporated bioactive compounds. Such structural modifications could have important implications for the functional properties of the resulting complexes, including their digestibility, thermal stability, and potential health benefits when used in food applications.

#### 3.3.2. Scanning Electron Microscopy (SEM)

**Figure 2:**
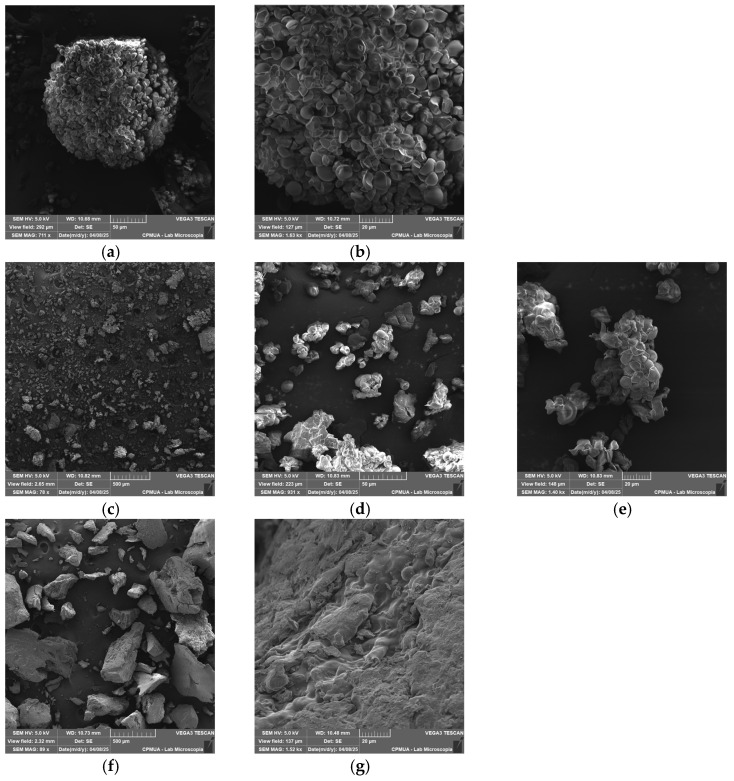
Scanning Electron Microscopy (SEM) images of native lobeira starch and starch–mirindiba extract complexes. (**a**) native lobeira starch (711 x), (**b**) native lobeira starch (2.47 kx), (**c**) starch complex with hydrophilic mirindiba extract (78 x), (**d**) starch complex with hydrophilic mirindiba extract (931 x), (**e**) starch complex with hydrophilic mirindiba extract (1.40 kx), (**f**) starch complex with lipophilic mirindiba extract (89 x), (**g**) starch complex with lipophilic mirindiba extract (1.52 kx). Magnifications correspond to those indicated in the scale bars of each micrograph.

SEM analysis revealed that native lobeira starch granules displayed a homogeneous distribution, with smooth, well-defined oval to irregular structures, typical of unmodified starches (Figure 2a,b). These morphological features are consistent with those reported by Carvalho et al. [7], who described *S. lycocarpum* starch granules as predominantly spherical to ellipsoidal with heterogeneous sizes. The absence of fissures and the integrity of granule boundaries observed here confirm the preservation of the native structure and the efficiency of the extraction process.

The well-defined morphology of the native starch serves as a baseline for evaluating structural modifications induced by complexation with mirindiba extracts. After the interaction with the hydrophilic extract, distinct morphological changes became apparent, including partial surface erosion and the appearance of amorphous deposits on the granule surfaces (Figure 2c–e). These alterations suggest that phenolic compounds were adsorbed onto the starch matrix, promoting physical reorganization of the granule surface. Such morphological modifications are consistent with previous observations in starch–neohesperidin systems, in which phenolic hydroxyl groups form hydrogen bonds with starch molecules, disrupting native intermolecular interactions and leading to looser, less compact structures [42]. Given the significant TPC concentration in the hydrophilic extract of mirindiba, it is plausible that similar interactions occurred, with the abundance of phenolic compounds intensifying the loosening effect on the starch network.

While interaction with the hydrophilic extract mainly led to surface adsorption and mild granule erosion, complexation with the lipophilic extract resulted in more profound structural rearrangements. In this case, the granules exhibited irregular and partially fused morphologies, with lamellar fragments and compacted regions (Figure 2f,g). These changes are consistent with the formation of inclusion-type complexes between amylose helices and lipophilic molecules, such as carotenoids, indicating stronger intermolecular interactions and partial disruption of the native starch architecture. The formation of a high number of microporous morphologies indicates the inclusion of carotenoids into the helical cavity of starch, as observed in V-type starch–lutein complexes [11].

#### 3.3.3. X-Ray Diffraction (XRD)

**Figure 3:**
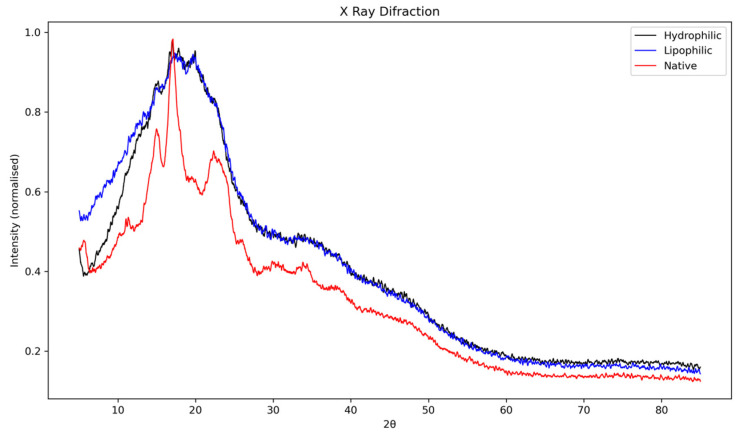
X-ray diffraction (XRD) patterns of native lobeira (*S. lycocarpum*) starch, starch complexed with hydrophilic extract of mirindiba (*Buchenavia tomentosa* Eichler), and starch complexed with lipophilic extract of mirindiba (*Buchenavia tomentosa* Eichler).

The X-ray diffraction patterns of native lobeira starch and its complexes with mirindiba extracts are shown in Figure 3. The native starch exhibited the typical semicrystalline profile of unmodified starches, with well-defined diffraction peaks between 15° and 23° (2θ). The reflections correspond mainly to the ordered lamellar regions associated with amylopectin and are consistent with a C-type crystalline pattern, previously reported for *S. lycocarpum* starch by Pascoal et al. [37], who identified strong reflections at approximately 15.3°, 17.2°, 19.6°, and 24.4°, along with a broad band around 22.2° (2θ). Such a pattern represents a mixture of A- and B-type polymorphs, typically displaying a doublet near 17°–18° characteristic of A-type crystallinity, and an additional reflection close to 5°, related to the B-type component. In agreement with these observations, the diffractogram of native lobeira starch confirms the presence of stable, ordered crystalline domains, primarily derived from amylopectin, coexisting with amorphous regions attributed to amylose and less-organized amylopectin fractions.

Complexation with the hydrophilic extract of *B. tomentosa* led to noticeable alterations in the native diffraction pattern. The characteristic peaks between 15° and 23° (2θ) exhibited reduced intensity and partial overlap, indicating a decrease in molecular order and an increase in amorphous content. This suggests that phenolic compounds interact with the starch matrix via hydrogen bonding and surface adsorption, thereby partially disrupting the crystalline order. Such behavior is consistent with the formation of non-inclusion complexes, as previously described for starch–flavonoid systems [43], where direct hydrogen bonding and electrostatic interactions occur without generating detectable V-type reflections in the XRD profile. This structural reorganization supports the partial loss of crystallinity observed in the hydrophilic complex, without fully transforming the native starch polymorph.

In contrast, the lipophilic extract complex showed main reflections between 15° and 23° (2θ), characteristic of semicrystalline regions associated with amylopectin. Although these peaks remained detectable, their intensity and definition were markedly reduced compared with native starch, indicating a decrease in crystallinity and an increase in amorphous content. In the region between 20° and 30° (2θ), a broadening and partial overlap of peaks were observed, suggesting molecular rearrangement and partial loss of long-range order. This pattern aligns with the formation of V-type inclusion complexes, typical of amylose-lipid or amylose-carotenoid systems, where hydrophobic guest molecules are embedded within the single-helical amylose cavities, consistent with previous observations of V-type recrystallization and guest-molecule encapsulation in amylose-based system [44].

Similar structural behavior has been reported by Song et al. [11] for starch–lutein complexes, in which the interaction of carotenoids within the amylose helix promoted the B-type to V-type crystalline transition, characterized by reflections near 13° and 20° (2θ). The authors also noted that lutein encapsulation increased crystallinity by stabilizing single helices within the starch matrix, confirming that these interactions extend beyond surface adsorption. In agreement with these findings, the more diffuse and broadened pattern observed here suggests that carotenoids from mirindiba extract penetrated the amylose helices, forming hydrophobic inclusion complexes that reorganized the crystalline domains and enhanced thermal and structural stability.

#### 3.3.4. Thermogravimetric Analysis (TG) and Differential Scanning Calorimetry (DSC)

**Figure 4:**
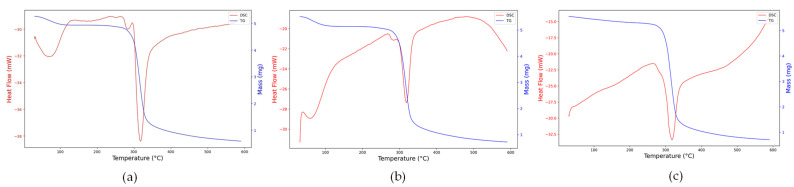
Thermogravimetric (TG, blue line) and differential scanning calorimetry (DSC, red line) curves of (**a**) native lobeira starch, (**b**) starch complexed with hydrophilic mirindiba extract, (**c**) starch complexed with lipophilic mirindiba extract.

Thermogravimetric (TG) and differential scanning calorimetry (DSC) analyses provided complementary insights into the thermal stability and degradation behavior of the native and complexed lobeira starches (Figure 4a–c).

The native starch exhibited the characteristic thermal behavior of semicrystalline polysaccharides (Figure 4a). The TG curve showed a single major degradation event between 270 and 340 °C, associated with glycosidic bond cleavage and volatilization of decomposition products. At the same time, the DSC trace revealed a sharp endothermic peak centered around 310 °C, characteristic of the rupture of semicrystalline domains and thermal decomposition of the polysaccharide matrix. An additional endothermic transition around 65 °C was also observed, corresponding to the evaporation of bound water and the onset of gelatinization, as previously reported for *S. lycocarpum* starch [36]. According to these authors, the relatively high gelatinization enthalpy (13 J/g) and low transition range (ΔT = 6.3 °C) are indicative of a homogeneous material rich in short amylopectin chains, features that explain the well-defined thermal response observed here.

For the hydrophilic mirindiba extract complex (Figure 4b), both TG and DSC profiles showed a reduction in enthalpy (−28 to −21 mW), confirming that the complexation decreased the molecular order of the starch. The persistence of the low-temperature endothermic event (<100 °C) suggests that phenolic compounds, rich in hydroxyl groups, enhanced the matrix’s water-binding capacity, promoting hydrogen-bond rearrangements without eliminating its hydrophilic character.

In contrast, the lipophilic extract complex (Figure 4c) showed no clear transition below 100 °C, indicating a reduced affinity for water due to the predominance of hydrophobic interactions with carotenoids and aliphatic chains. The main degradation peak shifted slightly to higher temperatures. It showed a smaller enthalpic range (−32.5 to −22.5 mW), indicating greater thermal stability and stronger inclusion-type (V-type) interactions between the starch helices and lipophilic molecules. This behavior aligns with the trend reported by Song [11], in which hydrophobic guest molecules restrict polymer mobility and delay the onset of decomposition.

Collectively, these thermal transitions confirm that complexation with hydrophilic or lipophilic bioactive extracts modifies the crystalline–amorphous balance of lobeira starch, influencing its interaction with water and overall thermal stability, key parameters for its potential application as a carrier matrix for bioactive compounds.

### 3.4. Biological Activity of Starch and Starch–Extract Complexes

#### 3.4.1. Cell Viability

**Figure 5:**
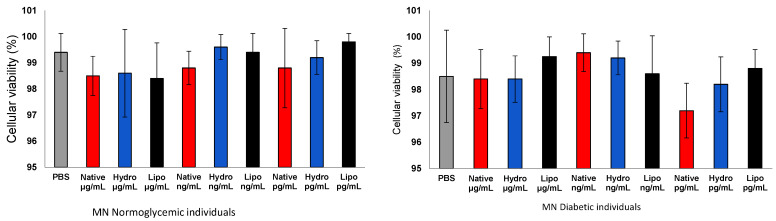
Cell viability of human peripheral blood mononuclear cells (MN) from normoglycemic and diabetic individuals after exposure to control, native starch, native starch complexes with hydrophilic mirindiba extract, and starch complexes with lipophilic mirindiba extract at concentrations of μg/mL, ng/mL, and pg/mL. Results are expressed as mean ± standard error. Significant differences among treatments were determined by ANOVA (*p* < 0.05).

The treatment of human mononuclear cells (MN) with native lobeira starch and starch–mirindiba extract complexes (hydrophilic and lipophilic) at different concentrations (μg/mL, ng/mL, and pg/mL) did not significantly affect cell viability (Figure 5) In both normoglycemic and diabetic groups, all treatments maintained a viability above 97%, comparable to the PBS control. These results indicate the high biocompatibility and low cytotoxicity of both native starch and starch–extract complexes, supporting their safety for further biological applications.

#### 3.4.2. Superoxide Anion Production (O_2_^•−^)

**Figure 6:**
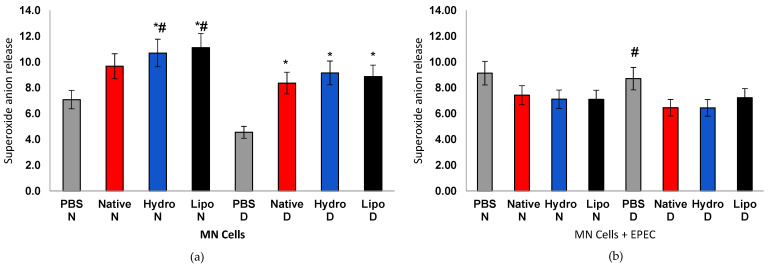
Superoxide anion release in human peripheral blood mononuclear cells (MN) from normoglycemic (N) and diabetic (D) individuals after treatment with native starch (Native), starch complexed with hydrophilic extract (Hydro), and starch complexed with lipophilic extract (Lipo), (**a**) in the absence and (**b**) presence of enteropathogenic *Escherichia coli* (EPEC). Data are expressed as mean ± standard error. * significant difference between untreated and treated cells within the same sample type and group (*t*-test, *p* < 0.05); # significant difference between groups (normoglycemic and diabetic) for the same treatment and sample type, in the absence or presence of EPEC (*t*-test, *p* < 0.05).

Superoxide anion generation varied according to metabolic condition and treatment (Figure 6a,b). In MN cells from normoglycemic individuals, exposure to both hydrophilic and lipophilic starch complexes led to a moderate increase in O_2_^•−^ production compared with non-stimulated controls, suggesting redox activation compatible with physiological adaptive signaling. This moderate ROS elevation can trigger antioxidant defenses, reflecting controlled redox signaling rather than oxidative stress, as reported for other adaptive cellular systems in which low ROS levels contribute to metabolic and immune homeostasis [45].

In contrast, MN cells from diabetic individuals, the marked increase in superoxide likely reflects an unbalanced redox state associated with hyperglycemia. Excessive antioxidant defenses, as previously described in hyperglycemia-induced mitochondrial reactive oxygen species (mtROS) overproduction and diabetic oxidative complications [46]. However, under *E. coli* (EPEC) incubation, cells from normoglycemic individuals maintained stable O_2_^•−^ levels across treatments, whereas cells from diabetic individuals showed a significant reduction compared with PBS controls. This pattern suggests that, while complexes can transiently stimulate redox signaling in resting cells, they act protectively under stress conditions by attenuating excessive ROS generation and restoring homeostasis.

#### 3.4.3. Superoxide Dismutase (SOD) Activity

**Figure 7:**
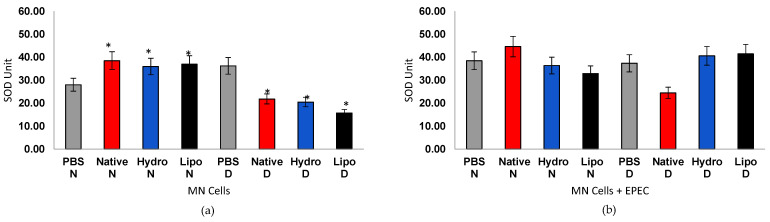
Superoxide dismutase (SOD) activity in human peripheral blood mononuclear cells (MN) from normoglycemic (N) and diabetic (D) individuals after treatment with native starch (Native), starch complexed with hydrophilic extract (Hydro), and starch complexed with lipophilic extract (Lipo), (**a**) in the absence and (**b**) presence of enteropathogenic *Escherichia coli* (EPEC). Data are expressed as mean ± standard error. * Significant difference between untreated and treated cells within the same sample type and group (*t*-test, *p* < 0.05).

SOD activity showed opposite trends between physiological conditions (Figure 7a,b). In MN cells from normoglycemic individuals, both native starch and starch–extract complexes significantly increased SOD activity compared to the PBS group, evidencing reinforcement of antioxidant defense mechanisms. Conversely, in cells from diabetic individuals, overall SOD activity declined, consistent with the impaired redox response typical of hyperglycemic conditions. Nonetheless, the lipophilic complex displayed the smallest decrease, suggesting partial restoration of enzymatic activity. Under EPEC stimulation, cells from normoglycemic individuals treated with native starch exhibited a pronounced increase in SOD activity. In contrast, cells from diabetic individuals treated with starch–extract complexes showed reactivation of the enzyme compared with diabetic controls. These findings indicate that bioactive compounds from mirindiba may help reestablish the redox balance in compromised cells, supporting their immunomodulatory potential.

The increased SOD activity and partial restoration of antioxidant defenses observed in cells from diabetic individuals treated with starch–extract complexes may reflect a sustained redox modulation mediated by the gradual release of bioactive compounds from the starch matrix. Similar mechanisms have been proposed for starch–bioactive inclusion systems, in which phenolic compounds or lipophilic ligands are entrapped within amylose helices, thereby enhancing their stability and antioxidant activity [47,48]. However, unlike previous studies that relied solely on chemical antioxidant assays (e.g., DPPH or ABTS), the present ex vivo analysis directly demonstrates the cellular relevance of these complexes. This represents, to the best of our knowledge, the first evidence of starch–bioactive complexes modulating oxidative stress markers in human mononuclear cells, providing biological validation for their proposed antioxidant potential.

#### 3.4.4. Oxidative Stress Index (OI)

**Figure 8:**
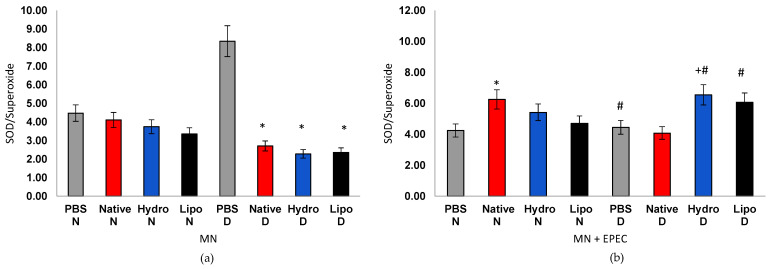
Oxidative index in human peripheral blood mononuclear cells (MN) from normoglycemic (N) and diabetic (D) individuals after treatment with native starch (Native), starch complexed with hydrophilic extract (Hydro), and starch complexed with lipophilic extract (Lipo), (**a**) in the absence and (**b**) presence of enteropathogenic *Escherichia coli* (EPEC). Data are expressed as mean ± standard error. * significant difference between untreated and treated cells within the same sample type and group (*t*-test, *p* < 0.05); # significant difference between groups (normoglycemic and diabetic) for the same treatment and sample type, in the absence or presence of EPEC (*t*-test, *p* < 0.05); + significant difference between treatments within the same group and sample type (ANOVA, *p* < 0.05).

The oxidative index (OI) integrates the interplay between oxidant production and antioxidant defense (Figure 8a,b). In cells from normoglycemic individuals, OI values remained stable (4.5) regardless of treatment, suggesting a balanced redox state. In cells from diabetic individuals, however, the control group exhibited markedly elevated ratios (8.0–9.5), confirming chronic oxidative stress. Treatments with native starch and, more prominently, with starch–extract complexes led to significant OI reductions, evidencing modulation of oxidative stress. In EPEC infection, cells from normoglycemic individuals showed a mild OI increase (5.5–6.5), indicating an adaptive antioxidant response. Cells from diabetic individuals, in turn, showed a pronounced decrease in OI in PBS controls (4.4), reflecting exhaustion of antioxidant defenses. Remarkably, both hydrophilic and lipophilic complexes partially restored the SOD/O_2_^• ^ratio (6.0–6.5), outperforming native starch and indicating recovery of redox homeostasis.

Altogether, the findings demonstrate that native and complexed lobeira starches modulate oxidative and antioxidant responses in human mononuclear cells, depending on both metabolic and infectious conditions. Cells from normoglycemic individuals exhibited a more adaptive redox behavior, characterized by moderate superoxide release and SOD activation, suggesting a functional balance between pro-oxidant signaling and compensatory antioxidant mechanisms.

In contrast, mononuclear cells from diabetic individuals showed altered responses, with elevated basal oxidative stress and reduced ability to adequately induce SOD activity in response to starch or bacterial challenge. This pattern reflects the intrinsic vulnerability of cells from individuals with diabetes to oxidative and inflammatory damage, a hallmark of chronic hyperglycemia.

Like any scientific investigation, this study has its limitations, which should be regarded as perspectives for advancement rather than restrictions. Our ex vivo results provide a consistent framework to understand how starch–lipophilic or –hydrophilic extract complexes from Cerrado fruits influence oxidative stress in human mononuclear cells. One limitation is that the analysis focused only on specific oxidative stress markers, without assessing other enzymatic or inflammatory pathways. While additional studies, including quantification of resistant starch and in vivo validation, are required to broaden these findings, the present evidence already demonstrates a direct connection between structural modifications of non-conventional starch and modulation of cellular antioxidant activity. Future research directions should address the digestion profile, bioaccessibility, and systemic effects of these complexes in diabetic models. In this context, the interplay among dietary immunomodulators, metabolism, and bacterial infection is highly complex, underscoring the need for personalized therapeutic strategies that account for both metabolic and immunological status.

## 4. Conclusions

The effects of lobeira (*S. lycocarpum*) starch complexed with hydrophilic and lipophilic extracts from mirindiba (*B. tomentosa*) were investigated through a multilevel approach encompassing chemical, structural, and biological analyses. The mirindiba extracts exhibited high concentrations of phenolic compounds and carotenoids, confirming their strong antioxidant capacity. Upon complexation, the structural and spectroscopic data (FTIR, XRD, TG, and SEM) revealed that these bioactive molecules interacted effectively with the starch matrix, forming inclusion-type complexes and promoting rearrangements in the crystalline and amorphous domains.

In biological assays, the complexes exhibited high biocompatibility, maintaining cell viability above 97% in both normoglycemic and diabetic groups. The modulation of oxidative and antioxidant responses was dependent on metabolic and infectious conditions. Mononuclear cells from normoglycemic individuals exhibited a greater adaptive capacity, characterized by moderate superoxide generation and compensatory activation of SOD activity, indicating a functional balance between pro-oxidant stimuli and compensatory antioxidant mechanisms. In contrast, cells from individuals with diabetes showed an elevated basal oxidative stress profile and a reduced ability to activate antioxidant defenses, reflecting the inherent vulnerability of the hyperglycemic environment. Under *E. coli* (EPEC) challenge, the starch–extract complexes partially restored of antioxidant responses, mitigated oxidative stress, and demonstrated an adaptive immunomodulatory effect.

Altogether, these findings confirm that complexation of non-conventional starch with bioactive extracts from Cerrado fruits induces structural modifications linked to cellular redox modulation under distinct metabolic contexts. This dual functionality—structural stabilization and biological modulation—positions lobeira–mirindiba complexes as promising dietary immunonutrients capable of restoring antioxidant balance in diabetes and infection. Furthermore, this study provides the first ex vivo evidence of the capacity of starch–bioactive complexes to influence cellular oxidative homeostasis, extending their relevance beyond chemical characterization toward functional health applications.

Future research should extend these findings by exploring the digestibility, bioaccessibility, and immunomodulatory mechanisms of these starch–bioactive complexes within the broader Pan-Amazonian context. Such comparative studies across biomes will deepen our understanding of how biodiversity influences bioactive composition and functional potential in regional species such as *B. tomentosa* and *S. lycocarpum*.

## Figures and Tables

**Table 1:** Proximate composition of lobeira (*Solanum lycocarpum*) fruit.

Parameters	Concentration (g/100 g)
Moisture (wet basis)	79.42 ± 0.17
Proteins (wet basis)	7.08 ± 0.09
Lipids (wet basis)	0.67 ± 0.05
Ash (wet basis)	3.31 ± 0.03
Carbohydrates (wet basis)	9.52 ± 0.20
Starch yield (dry basis)	55.55 ± 0.30
Energetic value (kcal/100 g)	72.43

## Data Availability

The original contributions presented in this study are included in the article. Further inquiries can be directed to the corresponding author.
